# Aminooxyacetic acid hemihydrochloride inhibits osteoclast differentiation and bone resorption by attenuating oxidative phosphorylation

**DOI:** 10.3389/fphar.2022.980678

**Published:** 2022-09-30

**Authors:** Biao Yang, Yuangang Su, Shuai Han, Runfeng Chen, Ran Sun, Kewei Rong, Feng Long, Hailong Teng, Jinmin Zhao, Qian Liu, An Qin

**Affiliations:** ^1^ Guangxi Key Laboratory of Regenerative Medicine, Collaborative Innovation Centre of Regenerative Medicine and Medical BioResource Development and Application Co-constructed by the Province and Ministry, Guangxi Medical University, Nanning, Guangxi, China; ^2^ Research Centre for Regenerative Medicine, Orthopaedic Department, The First Affiliated Hospital, Guangxi Medical University, Nanning, Guangxi, China; ^3^ Guangzhou Medical University, Guangzhou, China; ^4^ Shanghai Key Laboratory of Orthopaedic Implants, Department of Orthopaedics, Shanghai Ninth People’s Hospital, Shanghai Jiao Tong University School of Medicine, Shanghai, China

**Keywords:** aminooxyacetic acid hemihydrochloride, osteoclast, oxidative phosphorylation, energy metabolism, reactive oxygen species

## Abstract

Osteoclasts undergo active metabolic reprogramming to acquire the energy needed during differentiation and bone resorption. Compared with immature osteoclasts, mature osteoclasts comprise higher levels of electron transport chain enzymes and more metabolically active mitochondria. Of all energy metabolism pathways, oxidative phosphorylation is considered to be the most efficient in supplying energy to osteoclasts. We found that the malate-aspartate shuttle inhibitor aminooxyacetic acid hemihydrochloride inhibits osteoclastogenesis and bone resorption by inhibiting exchange of reducing equivalents between the cytosol and the mitochondrial matrix and attenuating mitochondrial oxidative phosphorylation *in vitro*. The weakening of the oxidative phosphorylation pathway resulted in reduced mitochondrial function and inadequate energy supply along with reduced reactive oxygen species production. Furthermore, treatment with aminooxyacetic acid hemihydrochloride helped recover bone loss in ovariectomized mice. Our findings highlight the potential of interfering with the osteoclast intrinsic energy metabolism pathway as a treatment for osteoclast-mediated osteolytic diseases.

## Introduction

Continued remodeling of healthy bone tissue is dependent on the homeostasis of bone resorption and bone formation (Boyle et al., 2003; Kim et al., 2020). Excessive osteoclast formation and activation can disrupt bone remodeling homeostasis and result in various osteolytic diseases, including postmenopausal osteoporosis, periprosthetic osteolysis, and inflammatory bone destruction (Bi et al., 2017; Yang et al., 2021). Therefore, the development of new treatments targeting osteoclasts has always been a priority (Sun et al., 2020; Yao et al., 2021). Although there exist many therapeutic drugs targeting osteoclasts, they have serious side effects and their long-term treatment effects are not desirable (Khosla and Hofbauer, 2017).

The energy requirement is greater in the process of the receptor activator of nuclear factor-κB ligand (RANKL)-induced osteoclastogenesis and activation, and osteoclasts undergo active metabolic reprogramming to obtain the energy to meet this demand (Arnett and Orriss, 2018; Kubatzky et al., 2018; Park-Min, 2019). Fully differentiated osteoclasts showed higher of oxidative phosphorylation (OXPHOS) complex protein levels, mitochondrial numbers, and mitochondrial metabolic activity (Lemma et al., 2016; Taubmann et al., 2020). Adenosine triphosphate (ATP) produced by the mitochondrial OXPHOS pathway is considered the main energy source for meeting the energy requirements of osteoclasts (Li et al., 2020; Da et al., 2021). Furthermore, in the OXPHOS pathway, mitochondria use oxygen to generate ATP from organic molecules through the electron transport chain (ETC), and the reactive oxygen species (ROS) generated during this process can affect various signal transduction processes in the cells (Lee et al., 2011; Foo et al., 2022). Previous studies have demonstrated that ROS, as intracellular secondary messengers, play pivotal roles in osteoclastogenesis and function (Chen et al., 2019; Xian et al., 2021). The level of intracellular ROS increases in response to RANKL stimulation and promotes the nuclear factor of activated T cells c1 (NFATc1) activity, which further affects the expression of downstream osteoclast-specific genes (Agidigbi and Kim, 2019; Chen et al., 2020). Studies have shown that osteoclastogenesis is inhibited with reductions in the levels of RANKL-induced ROS (Kim et al., 2017; Ni et al., 2020). Moreover, relatively high levels of ROS disrupt the regulation of bone homeostasis by promoting bone resorption (Li et al., 2017).

Aminooxyacetic acid hemihydrochloride (AOAA) is an inhibitor of the malate-aspartate shuttle (MAS). AOAA has been reported to inhibit intracellular ATP levels and alter cell cycle in C6 glioma cells, and also reduce glycolysis rate, extracellular lactate and pyruvate levels (Wang et al., 2016). In addition, AOAA regulates the survival and energy metabolism of resting or activated microglia by effectively inhibiting MAS activity (Chen et al., 2015). As a reduced nicotinamide adenine dinucleotide (NADH) shuttle, MAS can exchange the reducing equivalents of NADH between cytosolic and mitochondria (McKenna et al., 2006; Borst, 2020). The NADH in mitochondria furnishes the reduced equivalent to the mitochondrial ETC to fuel OXPHOS to generate ATP and is also involved in antioxidant/oxidative stress, gene expression, calcium homeostasis, aging, and cell death (Cantó et al., 2015; Covarrubias et al., 2021). We reported that AOAA inhibited the entry of NADH into the mitochondria, thus attenuating OXPHOS. Inadequate energy supply led to restricted osteoclast differentiation and function. Reductions in mitochondrial function also led to decreased ROS production. Furthermore, AOAA treatment recovered bone loss in ovariectomized (OVX) mice. Overall, we aimed to assess if the treatment of osteolytic diseases is possible by interfering with osteoclast intrinsic energy metabolism.

## Materials and methods

### Media and reagents

AOAA was purchased from MedChemExpress (Shanghai, China; #CAS 2921-14-4; purity ≥98%), dissolved in phosphate-buffered saline (PBS; Beyotime Biotechnology, Shanghai, China) at a concentration of 20 mM at −20°C, and further diluted to working concentration using culture medium. Penicillin/streptomycin (P/S), the alpha modification of minimal essential medium (α-MEM), and fetal bovine serum (FBS) were obtained from Thermo Fisher Scientific (Scoresby, VIC, Australia). Recombinant mouse macrophage-colony stimulating factor (M-CSF) and RANKL were obtained from R&D Systems (Minneapolis, MN, United States). Primary antibodies for OXPHOS (ab110413) and c-Fos (ab134122) were purchased from Abcam (Cambridge, United Kingdom), whereas NFATc1 (sc-7294) was purchased from Santa Cruz Biotechnology (Dallas, CA, United States). Antibodies specific to β-actin (# 4970) and secondary antibodies were obtained from Cell Signaling Technology (Danvers, MA, United States). The antibody concentrations were determined following the manufacturer’s recommendations.

### Cell culture and osteoclastogenesis assays

Bone marrow cells from both lower extremities of 6-week-old mice were washed out under aseptic conditions using a 1-ml syringe and then centrifuged at 1500 RPM for 3 min in a 50-ml centrifuge tube. The supernatant was discarded and the cells were resuspended in complete α-MEM (α-MEM supplemented with 1% P/S and 10% FBS) containing 30 ng/ml M-CSF. The resuspended cells were transferred to T-75 culture flasks (Thermo Fisher Scientific) and incubated for 4 days at 37°C to obtain the required bone marrow-derived macrophages (BMMs) for the next experiment.

BMMs (7 × 10^3^ cells/well) were cultured in 96-well plates using complete α-MEM supplemented with 30 ng/ml M-CSF, 50 ng/ml RANKL, and AOAA (0, 0.2, 0.3, and 0.4 mM) for 6 days to obtain osteoclasts. Next, the culture medium was discarded and 50 μl of 4% paraformaldehyde (PFA Solarbio, Beijing, China) was added to each well for 25 min. Tartrate-resistant acid phosphatase (TRAP) staining was performed after three washings with PBS. Representative images of TRAP staining were acquired using Cytation 5 (BioTek Instruments, VT, United States), and the numbers of mature osteoclasts (nuclei ≥3) were counted using ImageJ 1.51 software (NIH, Bethesda, MD, United States).

### Cell viability assay

BMMs (7 × 10^3^ cells/well) were seeded into 96-well plates and cultured in complete α-MEM containing 30 ng/ml M-CSF and AOAA (0, 0.1, 0.2, 0.3, 0.4, and 0.5 mM) for 48 and 96 h. Next, 10 μl of the cell counting kit-8 (CCK-8; Sangon Biotech, Shanghai, China) reagent was added to each well, and the plate was incubated for 30 min away from light. The absorbance value at 450 nm was detected using the TriStar^2^ LB 942 multifunctional enzyme plate analyzer (Berthold Technologies, Germany).

### Podosome belt staining

Osteoclast formation was induced as described above with or without the addition of AOAA (0.3 and 0.4 mM) in 96-well plates. The fixed cells were dialyzed using 0.1% Triton X-100 (Solarbio) for 5 min and then blocked with 3% bovine serum albumin (BSA; Solarbio) for 30 min at ambient temperature. Osteoclasts were incubated with rhodamine phalloidin (for podosome belts, dilution 1:200 in 0.2% BSA) for 1 h at 37°C. Lastly, the nuclei were counterstained with DAPI (dilution 1:200 in 0.2% BSA) for 10 min away from light. Cytation 5 was used to obtain fluorescent images, and the area of the podosomal belt was quantified using ImageJ 1.51 software.

### Bone resorption assays

Small osteoclasts were first induced using RANKL in a 6-well plate. Subsequently, an equal number of cells were inoculated onto sterile bovine bone slices and cultured for 4 days with the intervention of AOAA (0, 0.3, and 0.4 mM). The bovine bone slices were then processed for imaging under a scanning electron microscope using Regulus 8100 (HITACHI, Japan), and the area of bone resorption pits was quantitatively analyzed using ImageJ 1.51 software.

### RNA extraction and quantitative real-time PCR

BMMs (1.5 × 10^5^ cells/well in 6-well plates) were cultured in medium supplemented with 50 ng/ml RANKL and AOAA (0, 0.3, and 0.4 mM) for 6 days. The total RNA was subsequently extracted from the cells using TRIzol reagent (Thermo Fisher Scientific). cDNA strands were obtained from RNA using a reverse transcription kit (Thermo Fisher Scientific), and the SYBR Green Master (Roche, Basel, Switzerland) dye was used in the qPCR. The PCR cycling conditions on the LightCycler 96 system (Roche) for qPCR were as follows: 95°C for 10 min, followed by 55 cycles at 95°C for 15 s, 60°C for 15 s, and final extension at 72°C for 40 s. We used the 2^−ΔΔCT^ method to calculate the expression of target genes. Please see Table 1 for information on the primers used in this experiment.

**TABLE 1:** Primer information.

Gene	NCBI ID	Primer sequences (5–3′)	
		Forward	Reverse
*Nfatc1*	18,018	CGT​GTC​GGC​AAA​GGA​GAG​G	CAC​ATA​ACT​GTA​GTG​TTC​TTC​CTC​G
*Acp5*	11,433	GAC​CCA​CCG​CCA​AGA​TGG​AT	CAC​GGT​TCT​GGC​GAT​CTC​TT
*Ctsk*	13,038	CCA​TAT​GTG​GGC​CAG​GAT​GAA	ACA​GAG​ATG​GGT​CCT​ACC​CG
*Dcstamp*	75,766	ACC​TAA​GCG​GAA​CTT​AGA​CAC​A	AGG​GCT​TCG​TGG​AAA​CAC​AT
*Calcr*	81,799	TCC​AAC​AAG​GTG​CTT​GGG​AA	CTT​GAA​CTG​CGT​CCA​CTG​GC
*Mmp9*	17,395	CTC​TCC​TGG​CTT​TCG​GCT​G	GCG​GTA​CAA​GTA​TGC​CTC​TGC
*Gapdh*	14,433	AAC​TTT​GGC​ATT​GTG​GAA​GG	ACA​CAT​TGG​GGG​TAG​GAA​CA

### Western blot analysis

BMMs (1.5 × 10^5^ cells/well in 6-well plates) were stimulated with RANKL and AOAA for the indicated time periods; then, the cells were lysed using radioimmunoprecipitation assay buffer (Solarbio) for 30 min at 4°C to harvest total protein. Loading buffer (Beyotime Biotechnology; 1:4) was added to the extracted protein, which was subjected to 10% sodium dodecyl sulfate-polyacrylamide gel electrophoresis (Epizyme Biotech, Shanghai, China). The separated proteins were transferred onto nitrocellulose membranes (Bio-Rad Laboratories, CA, United States) and blocked with 5% skim milk for 2 h at room temperature. Afterwards, the protein-containing membrane was incubated with primary antibody for 12 h in a small black box at 4°C. Next, the primary antibody was washed off, and the membrane was incubated with the fluorescent secondary antibody for 1 h at 37°C. Finally, images of the protein bands were acquired using the Odyssey near-infrared fluorescence imaging scanner (LI-COR Biosciences, Lincoln, NE, United States), and the grayscale values of the protein bands were calculated using ImageJ 1.51 software.

### Measurements of nicotinamide adenine dinucleotide, NAD^+^, and Adenosine triphosphate

The contents of intracellular ATP and intracellular NAD (including NADH and NAD^+^) were detected using an enhanced ATP assay kit and the NAD^+^/NADH assay kit (Beyotime Biotechnology). Briefly, the kits were used for detection after the BMMs (1 × 10^6^ cells/well in 6-well plates) were cultured in complete α-MEM containing 30 ng/ml M-CSF, 50 ng/ml RANKL, and AOAA (0.4 mM) for 48 h. We calculated the concentration of NAD according to the reagent dealer’s instructions. The luminescence (RLU) was detected using the TriStar^2^ LB 942 multifunctional enzyme plate analyzer.

### Measurements of intracellular reactive oxygen species, mitochondrial reactive oxygen species and mitochondrial membrane potential

The levels of intracellular ROS, Mitochondrial ROS (mtROS) and Mitochondrial Membrane Potential (MMP) were measured using the reactive oxygen species assay kit (Biosharp, Beijing, China), MitoSOX Red dye (Yeasen Biotechnology, Shanghai, China), and Mito-Tracker Red CMXRos dye (Beyotime Biotechnology). In brief, BMMs (7 × 10^3^ cells/well in 96-well plates) were cultured in complete α-MEM containing 30 ng/ml M-CSF, 50 ng/ml RANKL, and AOAA (0, 0.3, and 0.4 mM) for 48 h. The above kit/dyes were then used for the purpose of detection following the manufacturer’s instructions. Three microscopic field images were captured randomly using Cytation 5 and quantified using ImageJ 1.51 software.

### Generation of an ovariectomized mouse model and treatment

The animal study was reviewed and approved by The Animal Ethics Committee of Guangxi Medical University (No. 202006021). Twenty-four C57BL/6J mice (females, 11-week-old) were randomly divided into four groups containing six mice each: sham (injected PBS), OVX + PBS (injected PBS), OVX + estrogen (injected 100 ng/kg E_2_), and OVX + AOAA (injected 5 mg/kg AOAA). The mice were anesthetized with 3% tribromoethanol; next, both the ovaries of all mice except those in the sham group were surgically excised. A week later, the mice received intraperitoneal injections of PBS, E_2_, or AOAA every 2 days for 6 weeks.

### Enzyme-linked immunosorbent assay

The blood of the mice was collected from the eyeballs before sacrifice, and serum was then separated from the blood. The levels of serum PⅠNP and CTXⅠ were detected using enzyme-linked immunosorbent assay kits (Sangon Biotech).

### Micro-CT scanning and analyses

The tibias of mice were collected and fixed in 4% PFA at the end of the animal experiments. The left tibias were scanned using a μCT-100 scanner (Scanco Medical, Bassersdorf, Switzerland) with the following settings: 50 kV source voltage, 75 μa source current, and 9 μm pixel size. The 3D images of the tibias were then reconstructed and analyzed using Skyscan CT software (Bruker, Kontich, Belgium). Bone parameters of the region of interest below the tibial growth plate, which included bone volume/tissue volume (BV/TV), trabecular number (Tb.N), trabecular thickness (Tb.Th), and trabecular spacing (Tb.Sp) were assessed.

### Histological analyses

After micro-CT scanning, the fixed left tibias were decalcified using JYBL-Ⅰ decalcification solution (Solarbio), dehydrated, embedded into paraffin, and cut into 3-μm sections. Next, the bone tissue sections were subjected to TRAP and hematoxylin and eosin (H&E) staining. Images of stained sections were acquired at ×5 and ×20 magnifications under the DMi1 digital microscope (Leica, Germany) and analyzed using ImageJ 1.51 software. The liver and kidney were dehydrated, embedded in wax, and sliced for H&E staining. Representative images of the kidney and liver were obtained at ×10 and ×40 magnifications using the DMi1 digital microscope.

### Statistical analyses

Each experiment was repeated at least thrice. Statistical significance was calculated using one/two-way ANOVA or Student’s *t*-test, and *p* < 0.05 was considered to be significant.

## Results

### Aminooxyacetic acid hemihydrochloride suppresses RANKL-Induced osteoclastogenesis and osteoclast resorption function *in vitro*

**FIGURE 1:**
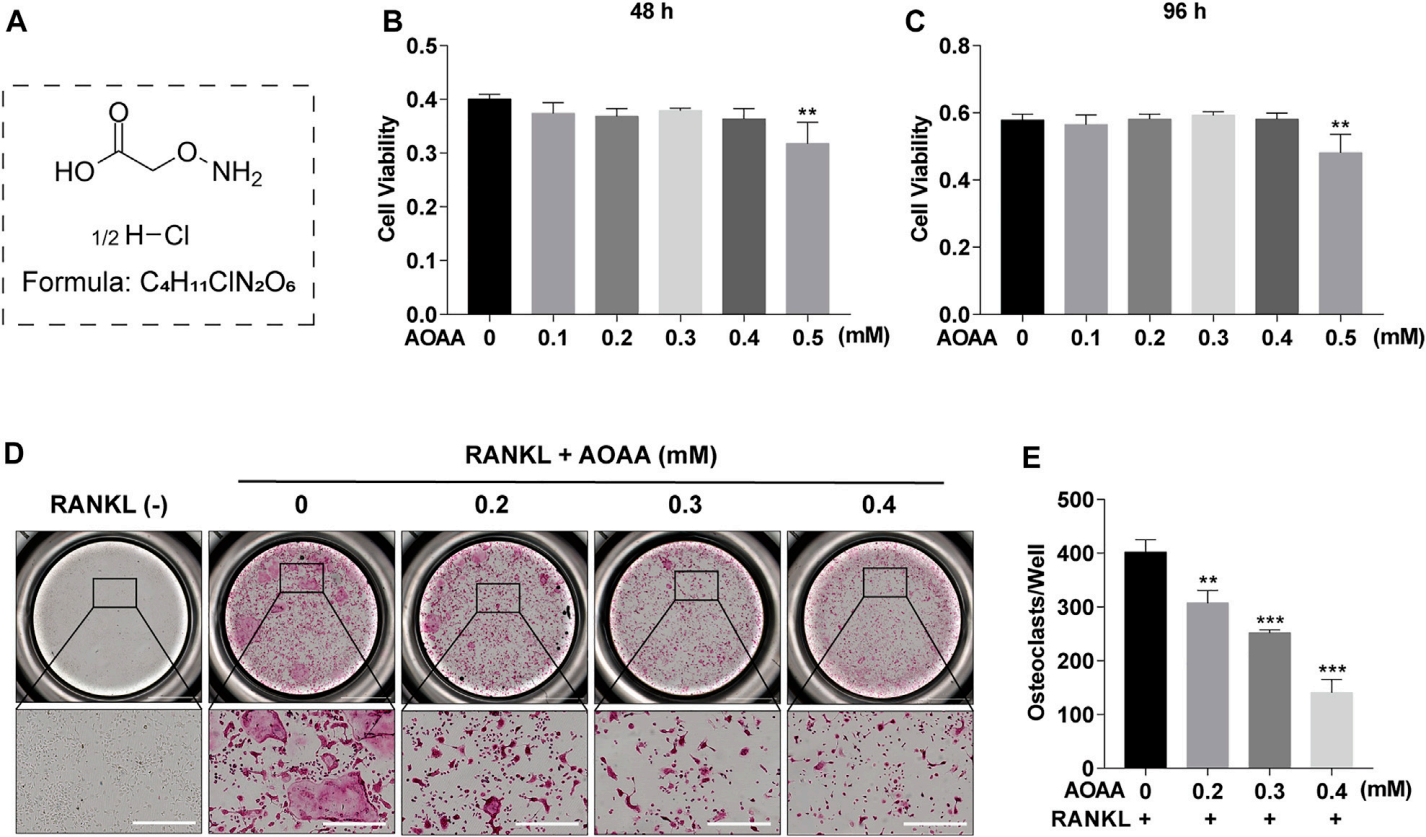
AOAA suppresses RANKL-induced osteoclastogenesis *in vitro* **(A)** The chemical structure of AOAA. **(B,C)** CCK-8 assay was used to detect the cell viability of BMMs after treatment with AOAA for 48 and 96 h. **(D)** Representative images of TRAP staining (scale bar = 500 µM). **(E)** Quantification of TRAP^+^ osteoclasts in each well (nuclei ≥3). All experimental data are expressed as mean ± SD (*n* = 3). **p < 0.01, ***p < 0.001 compared with that in the control group (AOAA, 0 mM). One-way ANOVA was used.

**FIGURE 2:**
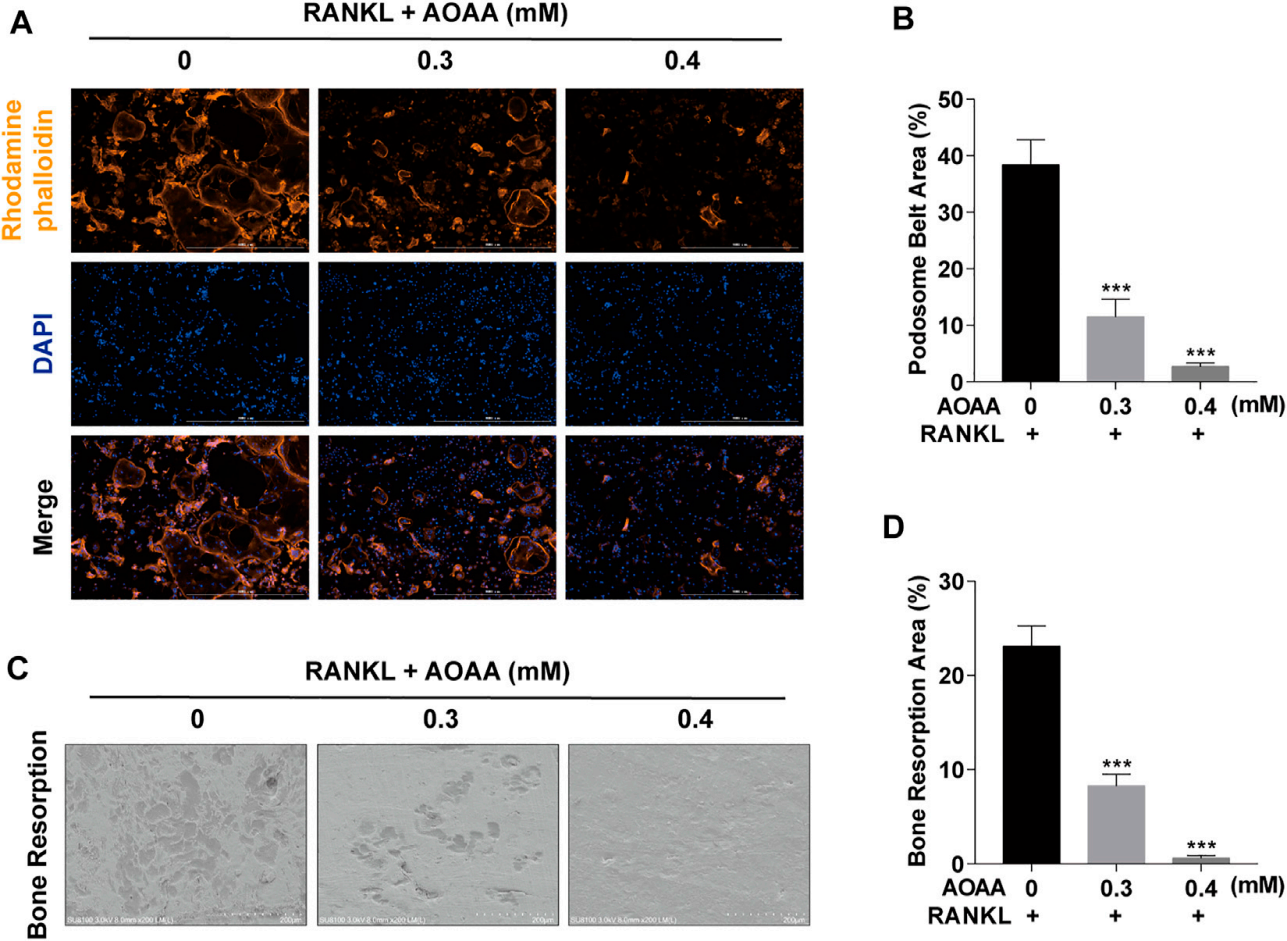
AOAA suppresses osteoclast resorption function *in vitro* **(A)** Representative images of podosome belt formation (orange, Rhodamine phalloidin) and nuclei (blue, DAPI) staining of osteoclasts (scale bar = 1000 µM). **(B)** Quantification of podosome belt area. **(C)** Representative images of osteoclast resorption function on bone slices (scale bar = 200 µM). **(D)** Quantification of bone resorption area. All experimental data are expressed as mean ± SD (*n* = 3). ***p < 0.001 compared with that in the control group (AOAA, 0 mM). One-way ANOVA was used.

The chemical structure of AOAA is shown in Figure 1A. The results of the CCK-8 assay after adding AOAA to co-culture BMMs for 48 and 96 h showed that AOAA at ≤ 0.4 mM had no effect on the cellular activity and proliferation of BMMS (Figures 1B,C). The results of *in vitro* TRAP staining revealed that osteoclast formation was inhibited after the addition of different concentrations of AOAA. A smaller area and fewer number of osteoclasts were formed after the incorporation of AOAA, compared with that in the 0 mM group (Figures 1D,E). The formation of an F-actin-based podosome belt, on which bone resorption depends, was inhibited, and the area was significantly reduced under the intervention of AOAA (Figures 2A,B). Therefore, we further inoculated small osteoclasts on sterile bovine bone pieces and observed the effects of AOAA on bone resorption. As shown in Figures 2C,D, abundant resorption pits appeared on the bone pieces upon the addition of RANKL only; moreover, the bone resorption function declined with the incorporation of AOAA and the area of resorption pits was significantly reduced. These results suggest that AOAA plays a role in resisting osteoclastogenesis and osteoclast resorption.

### Aminooxyacetic acid hemihydrochloride inhibits osteoclast-specific gene expression

**FIGURE 3:**
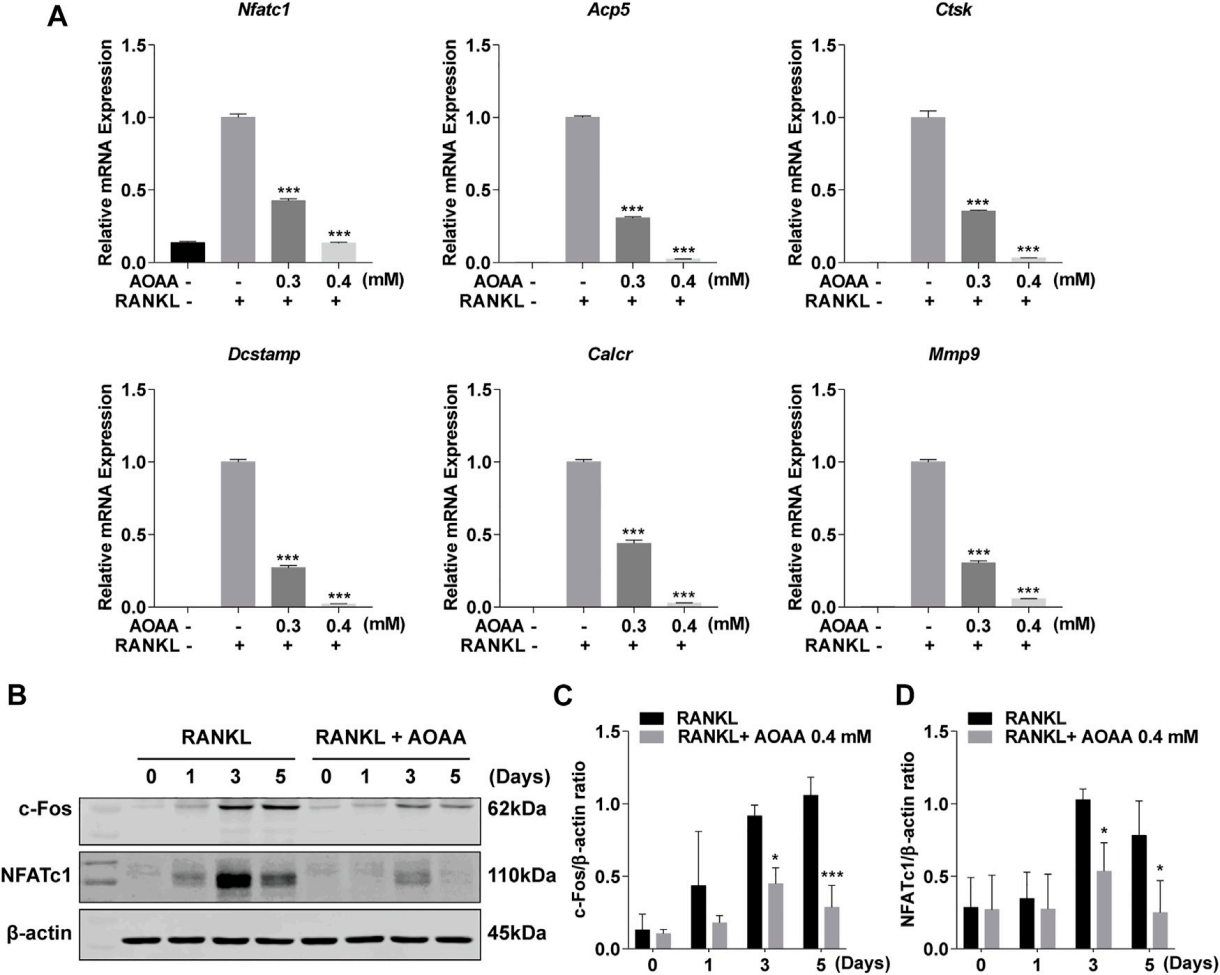
AOAA inhibits osteoclast-specific gene expression. **(A)** The osteoclast-specific gene expression of *Nfatc1*, *Acp5*, *Ctsk*, *Dcstamp*, *Calcr*, and *Mmp9* were quantitatively detected by qPCR (compared with the AOAA^−^ RANKL^+^ group). One-way ANOVA was used. **(B)** Representative western blot images of the effects of AOAA on NFATc1 and c-Fos expression. The leftmost bands of western blot represent the molecular weight marker. **(C,D)** Quantification of the ratios of band intensity of c-Fos and NFATc1 relative to β-actin (compared with RANKL-induced group at the same time point), two-way ANOVA was used. All experimental data are expressed as mean ± SD (*n* = 3). *p < 0.05, ***p < 0.001.

Considering the effect of AOAA on osteoclast phenotype and function, we used qPCR to detect changes in the expression levels of osteoclast-related genes, including *Nfatc1*, acid phosphatase 5 (*Acp5*), cathepsin K (*Ctsk*), dendrocyte expressed seven transmembrane protein (*Dcstamp*), calcitonin receptor (*Calcr*), and matrix metalloproteinase 9 (*Mmp9*). The intervention of AOAA diminished the stimulatory effect of the previous RANKL, thereby downregulating the expression of these genes (Figure 3A). In addition, western blot bands demonstrated increases in the protein expression of NFATc1 and c-Fos at days 3 and 5 in response to RANKL stimulation; however, AOAA counteracted the effect of RANKL and suppressed their expression (Figures 3B–D).

### Aminooxyacetic acid hemihydrochloride attenuates mitochondrial oxidative phosphorylation and reduces adenosine triphosphate production

**FIGURE 4:**
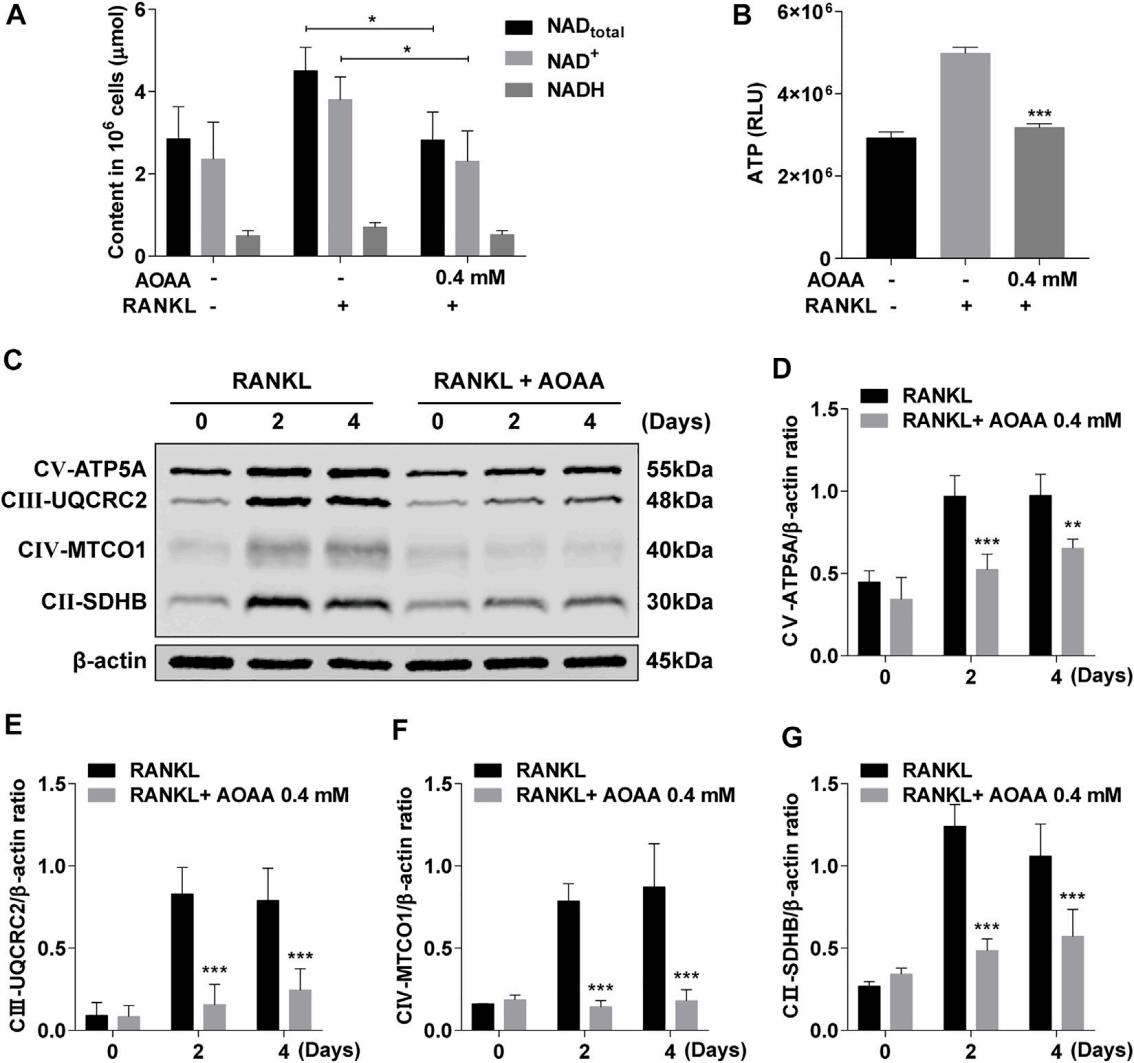
AOAA attenuates mitochondrial OXPHOS and reduces ATP production. **(A)** The relative levels of NAD^+^ and NADH were detected using the NAD^+^/NADH assay kit. Student’s *t*-test was used. **(B)** The relative levels of ATP were detected using the enhanced ATP assay kit (compared with AOAA^−^ RANKL^+^ group). One-way ANOVA was used. **(C)** Representative western blot images of the effects of AOAA on mitochondrial OXPHOS complexes (CⅤ-ATP5A, CⅢ-UQCRC2, CⅣ-MTCO1, CⅡ-SDHB). **(D–G)** Quantification of the ratios of band intensity of CⅤ-ATP5A, CⅢ-UQCRC2, CⅣ-MTCO1, and CⅡ-SDHB relative to β-actin (compared with RANKL-induced group at the same time point), two-way ANOVA was used. All experimental data are expressed as mean ± SD (*n* = 3). *p < 0.05, **p < 0.01, ***p < 0.001.

As an inhibitor of MAS, AOAA inhibited the exchange of reducing equivalents between the cytoplasm and the mitochondrial matrix, which resulted in altered levels of NAD in the cell (Figure 4A). The increase in the levels of intracellular total NAD and ATP was caused by the increased energy supply required for RANKL-induced osteoclastogenesis compared with that for BMMs; however, the levels decreased under AOAA treatment (Figures 4A,B). The expression of the OXPHOS complex protein increased significantly after 2 and 4 days of RANKL stimulation, but the protein expression declined considerably after the addition of AOAA during the same time period (Figures 4C–G). Thus, AOAA inhibits the exchange of reducing equivalents between the cytoplasm and the mitochondrial matrix by inhibiting MAS, which reduces the feedstock of mitochondrial OXPHOS, attenuates the level of OXPHOS, and inhibits energy production.

### Aminooxyacetic acid hemihydrochloride inhibits RANKL-induced reactive oxygen species and attenuates mitochondrial function

**FIGURE 5:**
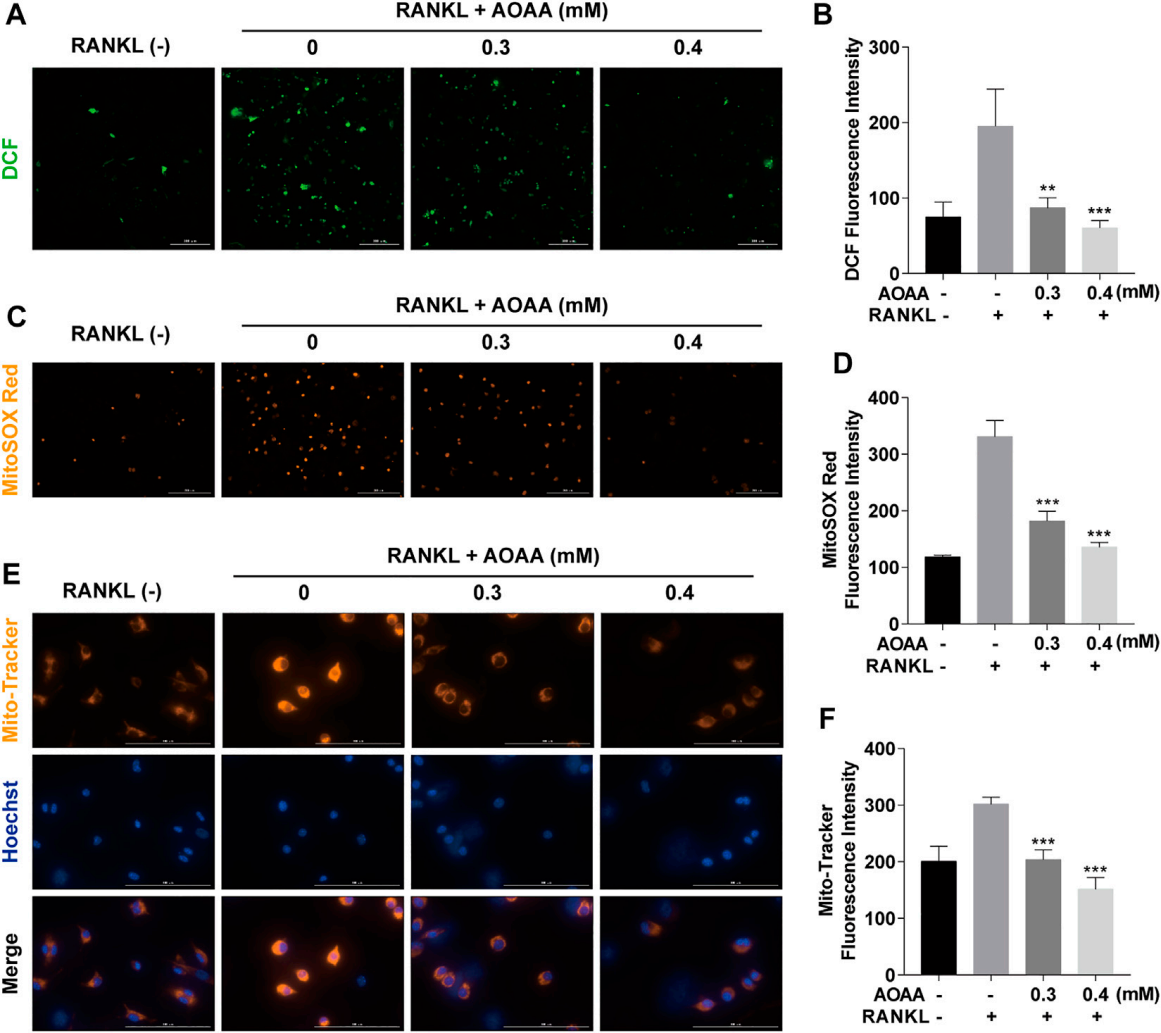
AOAA inhibits RANKL-induced ROS and attenuates mitochondrial function. **(A)** Representative fluorescence images of RANKL-induced ROS (green, DCF, scale bar = 300 µM). **(B)** Quantification of the DCF fluorescence intensity per field. **(C)** Representative fluorescence images of mitochondrial ROS (orange, MitoSOX Red, scale bar = 200 µM). **(D)** Quantification of the MitoSOX Red fluorescence intensity per field. **(E)** Representative fluorescence images of mitochondrial membrane potential (orange, Mito-Tracker) and nuclei (blue, Hoechst, scale bar = 100 µM). **(F)** Quantification of the Mito-Tracker fluorescence intensity per field. All experimental data are expressed as mean ± SD (*n* = 3). **p < 0.01, ***p < 0.001 compared with that in the AOAA^−^ RANKL^+^ group. One-way ANOVA was used.

ROS are important regulator of osteoclasts. Compared with RANKL-only stimulation, AOAA treatment significantly reduced intracellular ROS production (Figures 5A,B). Because mitochondria are factories for ROS production, we further examined mtROS. As shown in Figures 5C,D, stimulation with RANKL resulted in the accumulation of mtROS, whereas AOAA intervention attenuated the RANKL stimulation. During the processes of respiration and oxidation, the mitochondria store the generated energy in the form of electrochemical potential energy in the inner mitochondrial membrane, leading to asymmetric distribution of proton plasma on both sides of the membrane to form MMP. Because osteoclast differentiation and activation are energy-intensive processes, the MMP of the mitochondria in osteoclasts was relatively increased, but MMP was decreased in the presence of AOAA (Figures 5E,F). Overall, AOAA inhibits ROS production by attenuating mitochondrial function.

### Aminooxyacetic acid hemihydrochloride treatment prevents bone loss in ovariectomized mice

**FIGURE 6:**
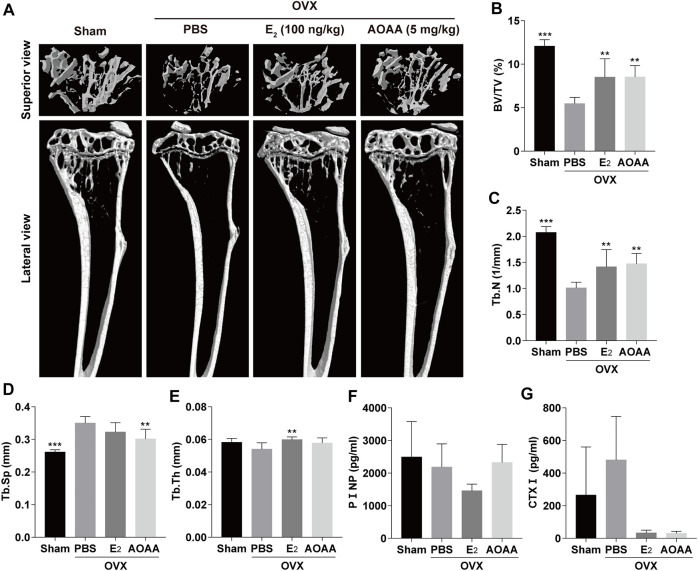
AOAA treatment prevents OVX-induced bone loss *in vivo*. **(A)** Representative micro-CT images of the tibia structures in each group. **(B–E)** Quantification of the parameters regarding the bone microstructure (including BV/TV, Tb.N, Tb.Sp, and Tb.Th) in each group (*n* = 6). **(F,G)** The relative levels of serum PⅠNP and CTXⅠ were detected using the enzyme-linked immunosorbent assay kits (*n* = 3). All experimental data are expressed as mean ± SD. **p < 0.01, ***p < 0.001 compared with that in the PBS group. One-way ANOVA was used.

Reconstructed micro-CT images of mouse tibia showed that ovariectomy significantly reduced bone mass and that AOAA treatment was effective in rescuing bone loss, comparable with that in the E_2_ group (Figure 6A). Bone parameters based on Micro-CT scans showed that AOAA improved BV/TV and Tb.N as well as lowered Tb.Sp, although Tb.Th was not significantly different compared with that in the PBS group (Figures 6B–E). By measuring the levels of CTXⅠ and PⅠNP, which are two biochemical indicators of bone metabolism in mice serum, we found that AOAA treatment greatly decreased the serum CTXⅠ level but did not significantly alter the PⅠNP levels compared with that in the PBS group (Figures 6F,G **)**.

**FIGURE 7:**
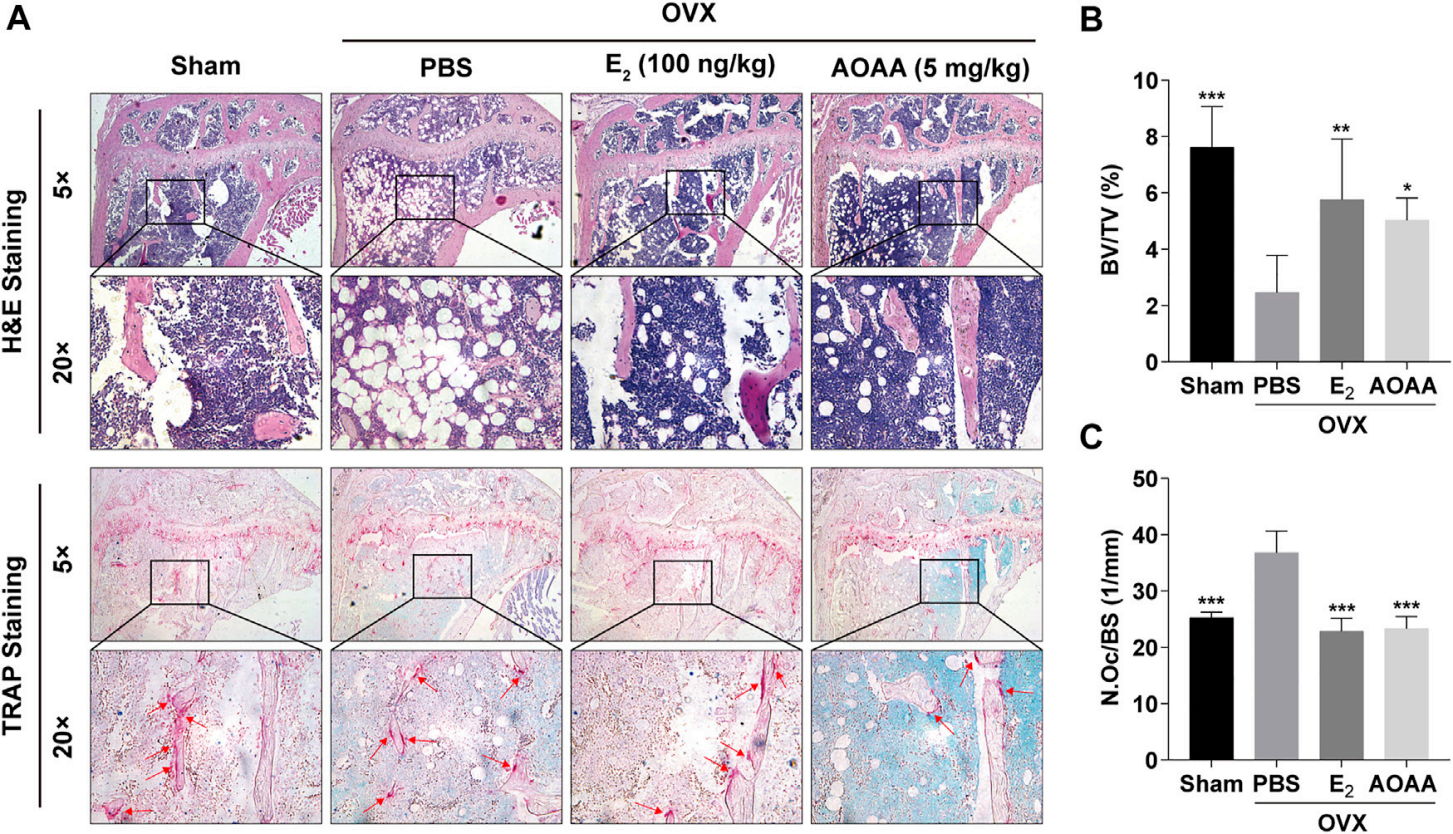
AOAA treatment reduces excessive osteoclastogenesis and resorption function in OVX mice. **(A)** Representative images of H&E and TRAP staining of decalcified bone sections in each group. **(B,C)** Quantitative analyses of BV/TV and N. Oc/BS in tissue sections. All experimental data are expressed as mean ± SD (*n* = 6). *p < 0.05, **p < 0.01, ***p < 0.001 compared with that in the PBS group. One-way ANOVA was used.

Quantification of H&E staining in mouse tibia showed that AOAA treatment recovered OVX-induced bone mass, which was consistent with the results of the micro-CT analysis (Figures 7A,B). Simultaneously, TRAP staining also showed that excessive osteoclastogenesis was inhibited in OVX mice under the intervention of AOAA (Figure 7A). A significant decrease was noted in the number of osteoclasts on the bone surface (N.Oc/BS, Figure 7C). Furthermore, AOAA had no visible toxic effects on the liver and kidney, which are the main metabolic organs, and did not alter their tissue morphology (Supplementary Figure 1).

## Discussion

Osteoclastogenesis and bone resorption that actively release protons to dissolve hydroxyapatite minerals are energy-consuming processes supported by high metabolic activity (Zhang et al., 2019; Guo et al., 2022). As osteoclast precursor cells fuse, the size, number, and metabolic activity of intracellular mitochondria gradually increase, and the osteoclasts undergo active metabolic reprogramming (Ishii et al., 2009; Roodman, 2009; Zeng et al., 2015). These abundant mitochondria are responsible for providing sufficient energy for osteoclasts. Although there exist several energy metabolism pathways in cells, the main energy supply for osteoclasts is derived from OXPHOS and glycolysis (Li et al., 2020; Guo et al., 2022). In addition, fatty acids and amino acids can serve as substrates to generate energy for osteoclasts through OXPHOS (Da et al., 2021). Therefore, OXPHOS has been greatly studied as the main metabolic pathway fulfill for energy requirements of osteoclasts and has been closely linked to other metabolic pathways (Lemma et al., 2016; Li et al., 2020; Taubmann et al., 2020). Consistent with that observed in other previous studies, the expression of OXPHOS complex protein increased, and the ATP content increased upon stimulation with RANKL (Lemma et al., 2016). Intracellular NAD levels were also elevated, as were the levels of MMPs. Appropriate MMP levels are a prerequisite for maintaining mitochondria for OXPHOS and ATP production (Gottlieb et al., 2003; Sanin et al., 2018). This supports the theory that osteoclasts are energy-dense bodies with high OXPHOS levels and metabolically active mitochondria. Wang and others' study found that pyruvate treatment completely counteracted the toxic effects of AOAA on C6 glioma cells, preventing AOAA-induced decreases in cell survival, intracellular ATP levels, and extracellular lactate levels (Wang et al., 2016). Kim et al. found that exposure of cultured cells to pyruvate, which promotes mitochondrial respiration and cooperates with RANKL to increase osteoclast production. Their results indicate that glucose metabolism during osteoclast differentiation is accelerated and that a metabolic shift towards mitochondrial respiration allows high ATP production and induces enhanced osteoclast differentiation (Kim et al., 2007). Coincidentally, the pyruvate concentration used in Wang and Kim’s study was consistent. These studies provide potential theoretical support and suggest that AOAA may inhibit osteoclasts by disrupting energy metabolism. The results of our study showed that the above-mentioned effects caused by RANKL stimulation were suppressed under the intervention of AOAA. This reveals the potential to inhibit osteoclastogenesis and function by interfering with osteoclast intrinsic energy metabolism pathways.

When we started our study, we considered how to determine the concentration range of AOAA. The study by Wang et al. found that AOAA treatment dose-dependently decreased the survival of C6 glioma cells, while not affecting the survival of primary astrocyte cultures (Wang et al., 2016). This suggests that there are concentration differences in different cell types. Based on their findings, we performed a cell viability assays in BMMs using the same concentration of AOAA. Because the vast majority of our experiments required AOAA intervention for at least 48 h, we further screened for safe thresholds by CKK-8 assays. In our TRAP staining experiments, the experimental results showed a relatively good gradient at the concentrations we chose, so the concentration range of AOAA was determined. However, the product information about AOAA (MedChemExpress) does not clearly indicate what the IC50 for inhibition of MAS is. The study by Korangath et al. found that transaminase activity in breast cancer cells decreased with increasing duration of AOAA (2 mM) action (Korangath et al., 2015). At 24 h, the transaminase activity decreased by about 50%. However, considering the particularity of breast cancer cells, the IC50 for inhibition of MAS may differ in other cells. Because the mitochondrial membrane is impermeable to NADH in the cytoplasm, the reducing equivalents need to rely on MAS for exchange between the cytoplasm and the mitochondrial matrix and mediate various biological functions, such as energy metabolism, mitochondrial function, and antioxidation (White and Schenk, 2012; Wang et al., 2016). AOAA is a most widely used inhibitor of MAS, which inhibits the shuttle by inhibiting aspartate aminotransferase (Chen et al., 2015). Aspartate aminotransferase catalyzes the transamination reaction that transfers amino groups from aspartate to alpha-ketoglutarate and regulates oxidative respiration and amino acid metabolism. Unfortunately, we did not dig deeper into the changes in other biochemical reactions in osteoclasts. We consider that the inhibition of osteoclasts by AOAA through the energy metabolic pathway is the main factor, but the alteration of other biochemical reactions should not be neglected. This part will be the focus of our subsequent study. The NADH in mitochondria is used as a raw material for OXPHOS to generate ATP through the ETC to meet the needs of the cells; a large amount of ROS are generated in this process (Ying, 2008; Foo et al., 2022). Our results revealed that intracellular NAD levels were reduced and ATP production was decreased under treatment with the MAS inhibitor AOAA. The reducing equivalents produced in the cytosol, mainly from glycolysis, will be prevented by AOAA from entering the mitochondria *via* MAS. This will affect glycolysis and force more pyruvate to be metabolized into lactate. Blocking MAS by AOAA will result in more pyruvate production of lactate and therefore less pyruvate is available to enter the mitochondria as substrate for the tricarboxylic acid (TCA) cycle. While NADH involved in mitochondrial respiration is mainly produced by the TCA cycle. This results in lower OXPHOS levels in the cell and reduced ATP production.

NFATc1 is the main transcription factor for osteoclast differentiation, and upstream c-Fos cooperates with it to induce the expression of osteoclast genes (Kim et al., 2014; Kwak et al., 2019). The blocked energy supply results in reduced transcriptional activity of c-Fos and NFATc1, further exacerbating the decrease in osteoclast-associated gene expression. Mature osteoclasts are surrounded by an F-actin-based podosome belt formed during the cytoskeletal reorganization of BMMs, and osteoclasts rely on the podosome belt to adsorb to the bone surface for osteolysis (Garbe et al., 2012; Lee, 2018). These high-energy-dependent osteoclast physiological activities are inhibited by AOAA. The OVX animal model is a classic model for simulating postmenopausal osteoclast over-formation and over-enhancement of function leading to osteoporosis (Lin et al., 2016; Fang et al., 2021). According to the results of our *in vitro* experiments, we further verified whether AOAA can affect osteoclasts *in vivo*. Both H&E staining and the micro-CT of the tibiae demonstrated that AOAA could protect bone volume in mice. TRAP staining revealed that AOAA reduced the number of osteoclasts, and the decrease in the serum CTXⅠ levels also suggested a reduction in bone resorption *in vivo* (Christenson, 1997). The current phase of osteoclast-inhibiting drugs, such as bisphosphonates, human monoclonal antibody to RANKL denosumab and the cathepsin K inhibitor odanacatib. Their rare side effects are a concern and the lack of clear evidence to support their long-term efficacy has led many patients to discontinue taking these drugs (Khosla and Hofbauer, 2017). Therefore, it is essential to continue developing effective drugs that do not cause these side effects and that improve patient acceptance and compliance. In our pre-experiments we observed that 10 mg/kg of AOAA caused a delay in healing of the surgical incision (about 7 days) and a slight loss of body weight in mice, which did not occur with 5 mg/kg of AOAA. Therefore, 5 mg/kg of AOAA was chosen for our formal animal experiments. In addition, H&E staining in our supplemental material shows no significant toxic effects on the kidney and liver at this dose of AOAA. It is important to note that virtually all cells have mitochondria and virtually all cells are dependent on MAS. Although our *in vivo* experiments show that AOAA can rescue bone loss in OVX mice, this is not bone-targeted and there may be potential effects of AOAA on other cells, which we need to continue to investigate in depth. Therefore, If AOAA is made to have targeting properties in subsequent studies or can be precisely localized in specific tissues and organs, this may bring us unexpected surprises.

In conclusion, our findings underscore the potential of interfering with osteoclast intrinsic energy metabolism pathways as a treatment for osteolytic diseases resulting from over-enhanced osteoclasts. Therefore, AOAA may serve as a new candidate or alternative therapy for osteoclast-related bone diseases.

## Data Availability

The original contributions presented in the study are included in the article/Supplementary Materials, further inquiries can be directed to the corresponding authors.
