# Supplementary material for: Aminooxyacetic acid hemihydrochloride inhibits osteoclast differentiation and bone resorption by attenuating oxidative phosphorylation
Source: Front Pharmacol. 2022 Sep 30;13:980678. doi: 10.3389/fphar.2022.980678 (PMC9561130; doi:10.3389/fphar.2022.980678)
Supplement: Supplementary file 1 [file Image1.PDF]

Supplementary Material

Supplementary Figures

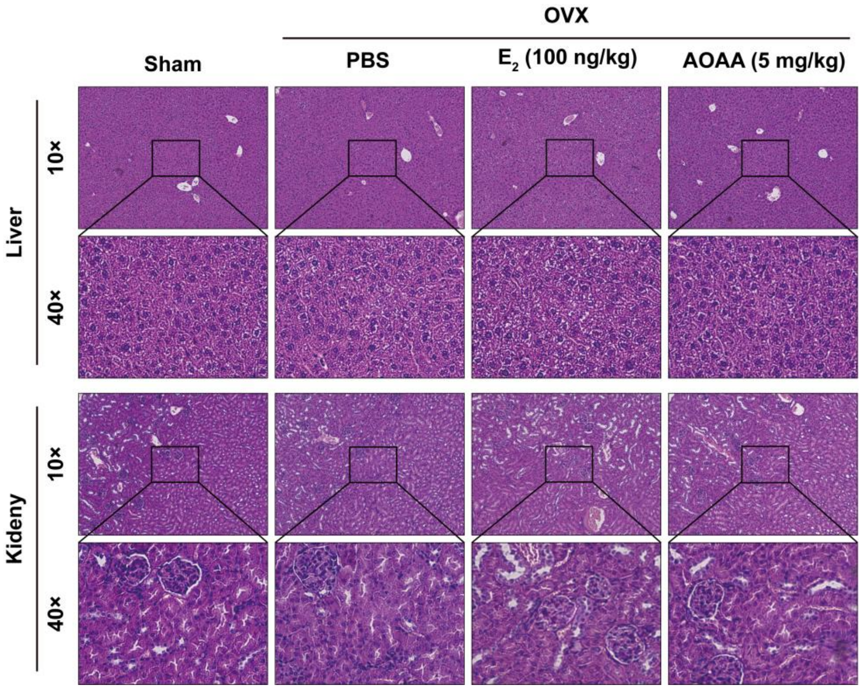

Supplementary Figure 1. AOAA has no obvious toxic effect on the liver and kidney in OVX mice. Representative images of H&E staining of liver and kidney in each group.
